# Thin film notch filters as platforms for biological image processing

**DOI:** 10.1038/s41598-023-31528-5

**Published:** 2023-03-18

**Authors:** Shaban B. Sulejman, Niken Priscilla, Lukas Wesemann, Wendy S. L. Lee, Jieqiong Lou, Elizabeth Hinde, Timothy J. Davis, Ann Roberts

**Affiliations:** 1 ARC Centre of Excellence for Transformative Meta-Optical Systems, School of Physics, The University of Melbourne, Melbourne, VIC 3010 Australia; 2 ARC Centre of Excellence for Transformative Meta-Optical Systems, Department of Electrical and Electronic Engineering, The University of Melbourne, Melbourne, VIC 3010 Australia; 3 School of Physics, The University of Melbourne, Melbourne, VIC 3010 Australia

**Keywords:** Biological physics, Photonic devices, Cellular imaging, Imaging and sensing, Phase-contrast microscopy, Biophotonics

## Abstract

Many image processing operations involve the modification of the spatial frequency content of images. Here we demonstrate object-plane spatial frequency filtering utilizing the angular sensitivity of a commercial spectral bandstop filter. This approach to all-optical image processing is shown to generate real-time pseudo-3D images of transparent biological and other samples, such as human cervical cancer cells. This work demonstrates the potential of non-local, non-interferometric approaches to image processing for uses in label-free biological cell imaging and dynamical monitoring.

## Introduction

Transparent objects, including most biological cells, interact weakly with light resulting in little contrast in conventional bright field microscopy. However, spatial variations in their morphology and optical properties introduce local phase variations onto light transmitted through them. In the simplest case, this can be characterized by a transmission function $O(x,y) \approx O_0 e^{i\varphi (x,y)}$. The approximately spatially invariant amplitude $O_0$ produces a featureless intensity image $|O(x, y)|^2 = |O_0|^2$, while the shape and refractive index information is contained in the phase function $\varphi (x,y)$. Such phase variations cannot be directly sensed by conventional cameras and therefore requires indirect detection. Popular optical phase visualization methods include Schlieren imaging^1^, as well as Zernike phase contrast^2^, dark field^3^ and differential interference contrast microscopy^4^. However, these can require expensive components or Fourier plane access that increases system complexity and size. Digital methods include ptychography^5–7^, the use of the transport of intensity equation^8–10^ or phase retrieval algorithms such as the Gerchberg-Saxton^11^ and Fienup algorithms^12^. However, these can be limited by their extensive computational requirements.

All-optical, object-plane image processing offers a non-interferometric and compact alternative for phase visualization. It is enabled by 2D space-invariant linear optical systems, such as thin films^13,14^, with angular responsivities that directly filter the spatial frequency of wavefields^15^. Unlike common computational or all-optical methods utilizing the classical $4f$-configuration^16^, it avoids optical phase information losses, energy consuming post processing and bulky configurations associated with accessing Fourier planes. The importance of compact optical systems for all-optical, object-plane image processing is motivated by the potential for integration into portable devices. This can have applications as diverse as mobile diagnostics, environmental monitoring and remote sensing.

To explain how a device exhibiting angular dispersion can perform image processing, we ignore any polarization effects for simplicity. In this case, the impact of object-plane Fourier filtering on the spatial frequency spectrum of the field can be described by an optical transfer function ${\mathscr {H}}(k_x, k_y)$^17^. By taking the $z$-axis as the optical axis, $k_{x}$ and $k_{y}$ denote the transverse spatial frequency components of the wave-vector $\vec {k} = (k_x, k_y, k_z)$ and $k_z = \sqrt{|\vec {k}|^2 - k_x^2 - k_y^2}$. The transfer function relates the processed output to the input field by the convolution theorem,

1 $$\begin{aligned} E_{ \text {out} }(x,y,z) = {\mathscr {F}}^{-1} \left\{ {\mathscr {H}}(k_x, k_y) \tilde{E}_{ \text {in} }(k_x, k_y; z) \right\} (x, y) \ , \end{aligned}$$

where ${\mathscr {F}}$ denotes the Fourier transform, $E$ represents any component of the electric field and $\tilde{E}_{\text {in}} = {\mathscr {F}}\left\{ E_{ \text {in} } \right\}$. For example, high-pass filters block low spatial frequencies to eliminate unscattered field components for edge detection^18^, which is fundamental to data compression^19^ and machine vision^20,21^. A notable sub-class is that of linear optical transfer functions, i.e. ${\mathscr {H}} \propto k_x$ or ${\mathscr {H}} \propto k_y$, that can compute the spatial derivatives, up to a multiplicative constant, of an incoming wavefield along the $x$ or $y$ direction, respectively. As a result, phase gradients can be mapped to intensity variations to permit phase visualization in the case of transparent samples^13^. The influence of polarization can be incorporated into this approach by utilizing a $2 \times 2$ transfer function dyadic tensor.

The angular sensitivity provides the mechanism for image processing through the correspondence between angles of incidence and spatial frequencies. This is given by representing the spatial frequency components in spherical coordinates^15^,

2 $$\begin{aligned} k_{x} & = k_{0} \sin \theta \cos \phi \\ k_{y} & = k_{0} \sin \theta \sin \phi \\ k_{z} & = k_{0} \cos \theta \;, \\ \end{aligned}$$

where $k_0=|\vec {k}|$ is the wavenumber, while $(\theta , \phi )$ are the polar and azimuthal propagation angles of plane waves with respect to the $z$-axis. Given that optical transfer functions represent plane wave responses in $k$-space, and that light can be decomposed into weighted plane waves by the spatial Fourier transform^15,22^, it follows that devices exhibiting angular dispersive transmission are capable of object-plane image processing.

Recently, meta-optical devices have attracted considerable attention as ultra-compact image processors^23^. For example, Zhou et al.^24^ employed photonic crystals for edge detection of organic samples, while Wesemann et al.^25^ obtained phase contrast images of human cancer cells using a resonant waveguide grating. Other approaches have involved Mie^26,27^ or Fano^28^ resonances, photonic spin-orbit coupling effects^29,30^ and bound states in the continuum^31^. With this rapidly growing interest in meta-optics, it is timely to consider other optical elements capable of performing an equivalent role. Earlier works have investigated various structures for edge detection such as volume hologram filters^32^, Fabry-Pérot etalons^33^, detuned interference filters^34,35^, acousto-optic modulators^36^ and gratings^37–39^. With the exception of Fourier plane phase contrast methods, the emergence of successful digital methods for image processing held back further progress in all-optical techniques. However, this is being revisited in the light of the rapid increase in data being generated and its associated impact on energy consumption and processing rates.

Here we demonstrate the use of a commercially available thin film spectral notch filter applied to phase contrast imaging of transparent samples. Notch filters are band-stop filters commonly employed in various types of spectroscopy to remove temporal frequencies over a specific range^40,41^. The angular dispersion of the filter’s rejection band is shown to produce a high-pass spatial frequency filter at the operating wavelength. The approach presented here is based upon introducing a phase bias by tilting the filter with respect to the optical axis to produce pseudo-three dimensional images similar to those obtained in differential interference contrast microscopy. The contrast generated is determined by the rotation axis and angle, while the field of view and resolution of images are determined by the specifications of the optical system. We demonstrate enhanced contrast imaging of wavefields introduced by a spatial light modulator and unstained biological samples. Our results confirm instantaneous phase contrast imaging without post-processing, permitting direct imaging with either a camera or the eye. The method provides an alternative all-optical approach for biological and other image processing with imaging capabilities comparable to conventional methods but using an off-the-shelf spectral band-stop filter. Their availability and the simplicity of the proposed approach makes it a fast and useful technique for obtaining insight into phase contrast images within any imaging system. It therefore has the potential for developments in machine vision, biological imaging and dynamical monitoring.

## Experimental results

### Device performance

**Figure 1:**
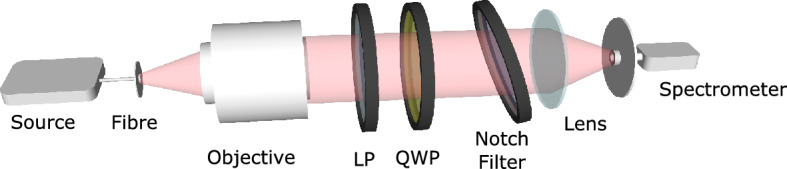
The experimental configuration used to capture the transmission spectra of the notch filter. Here, LP and QWP denote linear polarizer and quarter wave-plate, respectively. The schematic is not to scale.

**Figure 2:**
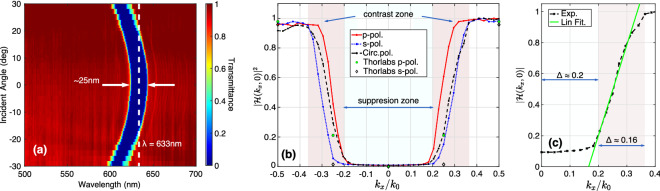
The experimental transmission response of the filter obtained by incrementally rotating the filter (**a**). The dashed line in (**a**) indicates the band-stop wavelength of 633 nm and the arrows indicate the width of the band-stop region at normal incidence. The modulus-square of the modulation transfer function along $k_y=0$ for various polarizations (**b**) is compared to data provided by the manufacturer. Linear fitting of the modulation transfer function for circular polarization in the contrast zone is given in (**c**).

The device investigated here is a commercially obtained notch filter (Thorlabs NF633-25), which has a specified central operating wavelength of 633 nm and band-width of 25 nm at normal incidence. The transmission spectra of the device as a function of angle of incidence were experimentally measured using the configuration in Fig. 1, with details provided in the “Methods” section. Collimated white light from a halogen lamp was passed through the notch filter at different angles of incidence and focused onto a spectrometer. The results (Fig. 2a) obtained using circularly polarized light are consistent with the manufacturer’s specifications. Meanwhile, the results for $p$- and $s$-polarized light are given in the supplementary information (§S1.1). These results confirm band-rejection at the operating wavelength with a band-width of approximately 25 nm, as well as blue-shifting of the band-stop region with increasing angle of incidence. Noting the correspondence between angles of incidence and spatial frequencies, it is found that the notch filter suppresses low spatial frequencies associated with near-normal incidence angles at the band-stop wavelength.

The modulation transfer functions $\vert{\mathscr {H}}(k_x, k_y)\vert$ for $p$-, $s$- and circular polarizations were mapped from the measured transmission spectra in Fig. 2a. Line profiles along $k_y=0$ (Fig. 2b) at the band-stop wavelength exhibit approximately polarization-insensitive, high-pass behaviour with a suppression zone of numerical aperture (NA) range $0.20$. This is a consequence of the blue-shifting of the band-stop wavelength with increasing angle of incidence. A significant feature of the device is the region of approximate linear dependence on $k_x$ from zero to near-unity transmission beyond the suppression zone. This is supported by linear fitting (Fig. 2c) of the modulation transfer function for circular polarization from $k_x/k_0 \approx 0.20$ to $0.30$, represented by the relation $A k_x/k_0 + B$. The polyfit function and curve fitting toolbox in MATLAB were used to obtain the fitting parameters and their error intervals as $A = 5.9 \pm 0.83$ and $B = -0.97 \pm 0.22$. Rotating the filter by ${12}^\circ$ about a line perpendicular to the optical axis shifts operation to $k_x/k_0 \approx \pm 0.20$ to access this region, referred to here as the contrast zone. This feature of the device permits visualization of phase variations in wavefields in the form of intensity changes.

### Phase contrast imaging—principles

To explain how the notch filter generates contrast related to the phase gradients in a wavefield, consider normally incident, monochromatic plane waves $E_s(x,y,z)$ of wavelength $\lambda$ and wavevector $\vec {k}$ propagating along the *z*-axis. The source illuminates a transparent sample with a transmission function $O(x, y) = O_0 e^{i \varphi (x, y)}$. Its visualization requires highlighting the phase shifts imparted onto light transmitted through it, which can be modelled as

3 $$\begin{aligned} E_{ \text {in} }(x,y) = O_0 e^{i \varphi (x, y)} E_s(x,y) \ , \end{aligned}$$

where we have ignored vector effects here for simplicity. Given the following property for the Fourier transform of derivatives,

4 $$\begin{aligned} {\mathscr {F}} \left\{ \frac{\partial E_{\text {in}}}{\partial x} \right\} (k_x, k_y)&= i k_x {\mathscr {F}}\{E_{\text {in}}(x, y)\}(k_x, k_y) \end{aligned}$$

5 $$\begin{aligned} {\mathscr {F}} \left\{ \frac{\partial E_{\text {in}}}{\partial y} \right\} (k_x, k_y)&= i k_y {\mathscr {F}}\{E_{\text {in}}(x, y)\}(k_x, k_y) \ , \end{aligned}$$

then a linear optical transfer function and Eq. (3) substituted into Eq. (1) produces the spatial derivative of $O(x,y)$,

6 $$\begin{aligned} \frac{\partial O(x,y)}{\partial x}&\approx i O_0 \frac{\partial \varphi (x,y)}{\partial x} e^{i\phi (x,y)} \end{aligned}$$

7 $$\begin{aligned} \frac{\partial O(x,y)}{\partial y}&\approx i O_0 \frac{\partial \varphi (x,y)}{\partial y} e^{i\phi (x,y)} \ . \end{aligned}$$

   This generates intensity images proportional to $| \partial \varphi (x,y)/\partial x |^2$ or $| \partial \varphi (x,y)/\partial y |^2$, with contrast created in regions where the phase is varying along the direction of differentiation.

The notch filter approximately produces the spatial derivative around an angular offset within the contrast zone. Operating near the edge of the contrast zone at a rotation angle of approximately ${12}^\circ$ removes low spatial frequency components, leaving only relatively large phase gradients corresponding to edges in images, as shown in the supplementary information (§S1.2). Therefore, the images produced would be comparable to those obtained in Schlieren imaging and dark field microscopy. Shifting operation to within the contrast zone at a rotation angle of ${14}^\circ$, corresponding to a normalized spatial frequency of $k_x/k_0 \approx 0.24$, preserves some of the background field permitting discrimination between positive and negative phase gradients. These manifest as different grayscale levels above or below the shifted $k$-space origin, respectively. Therefore, operating within the contrast zone enables phase visualization by mapping phase variations to changes in intensity. The presence of some unscattered components retains a non-zero intensity background that distinguishes this from dark field imaging. It instead creates pseudo-three dimensional contrast, similar to that seen in differential interference contrast microscopy, via information about the sign of the gradient. Moreover, the type of contrast can be controlled by changing the axis of rotation to visualize phase gradients along different directions. These form the key underpinnings of this article to describe the capacity of notch filters for phase visualization.

### Phase contrast imaging—experiment

**Figure 3:**
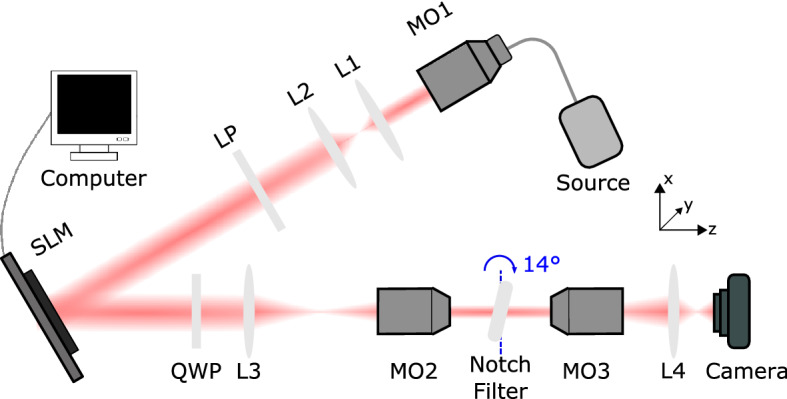
The experimental configuration for image processing with a spatial light modulator (SLM), where L and MO denote lenses and microscope objectives, respectively. The schematic is not to scale.

**Figure 4:**
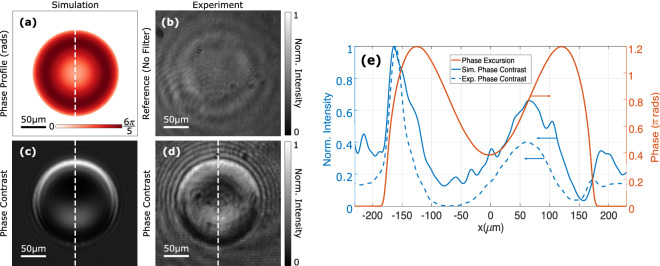
Image processing was performed on a transparent red blood cell (**a**) emulated by a spatial light modulator with a phase excursion of $6\pi /5$. An experimental control image obtained in the absence of the notch filter is given in (**b**), while simulated (**c**) and experimental (**d**) phase images produced by the notch filter demonstrate phase visualization. The intensity images (**b**)–(**d**) are normalized to their brightest pixels, while the line profiles along the dashed lines shown in (**a**), (**c**) and (**d**) are given in (**e**).

All-optical image processing was experimentally performed on various samples with the notch filter using circularly polarized, 635 nm laser light. Edge detection of amplitude samples was first demonstrated on a USAF resolution test target, with details and results provided in the supplementary information (§S1.2). Phase contrast imaging was then performed using the configuration in Fig. 3, which is detailed in the “Methods” section. A computer-controlled spatial light modulator, comprising 1920 $\times$ 1080 liquid crystal pixels of ${8}\,\upmu \hbox {m}$ pitch, emulated phase profiles of human red blood cells (Fig. 4a). These were modelled using the optical properties provided in Ref.^42^. Collimated light reflected from the spatial light modulator was imaged onto the notch filter, which was rotated within the focal plane between paired microscope objectives to ensure operation within the contrast zone of the device. The transmitted images were then captured by a camera. The field of view of the system was set by the aperture of the spatial light modulator and the optical components.

The results are presented in Fig. 4b–e, where simulated and experimental images are compared. An experimental control image (Fig. 4b) displays poor contrast, rendering the object virtually invisible as expected for weakly absorbing samples. This was obtained by capturing the image in the absence of the notch filter using the configuration in Fig. 3. A simulated phase image (Fig. 4c) was obtained using numerical simulations performed in Python, which is detailed in the “Methods” section. The corresponding experimental phase image (Fig. 4d) was obtained using the rotated notch filter in the configuration in Fig. 3. In both the simulated and experimental cases, the notch filter was rotated by an angle of ${14}^\circ$ to access the contrast zone. These preserved some unscattered contributions that are evident from their non-zero intensity backgrounds. In both cases, regions where a change in phase is present, i.e. $\nabla \varphi (x,y) \ne 0$, are revealed with an intensity contrast arising from otherwise invisible phase modulations.

The micrometre resolution, indicated by the scale bars on the images in Fig. 4a–d, was determined by the optical system. The resultant phase images additionally possessed the capacity to distinguish between positive and negative phase gradients, leading to the appearance of pseudo-three dimensional phase contrast. Furthermore, line profiles (Fig. 4e) exhibit intensity variations associated with phase variation introduced into the field by the sample. Some intensity artifacts can also be seen, such as those arising from amplitude variations in the field and ringing artifacts associated with the Gibbs phenomenon that surround the sample.

### Phase contrast microscopy

**Figure 5:**
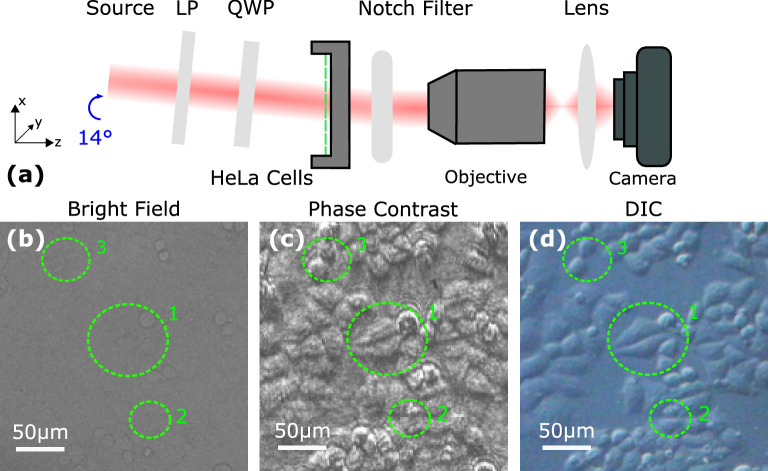
Biological phase imaging was performed using the experimental schematic in (**a**). The dashed line represents the HeLa cells within a petri dish and the schematic is not to scale. A bright field image of the HeLa cells obtained without the notch filter is given in (**b**). The corresponding phase contrast image obtained using the filter is given in (**c**) and a differential interference contrast image in (**d**). The dashed circles shown in (**b**)–(**d**) highlight regions where contrast was significantly enhanced for comparative purposes.

To illustrate potential applications, phase contrast microscopy was performed on biological samples with weak amplitude contrast. This was achieved using an inverted microscope configuration depicted in Fig. 5a and outlined in the “Methods” section. A cell line (HeLa) derived from human cervical cancer were used as the sample with preparation steps outlined in the “Methods” section. Their bright field image (Fig. 5b) obtained in the absence of the notch filter displays poor contrast, with little to no detail on the cellular features. However, placing the notch filter immediately beneath the sample enabled visualization of the phase variations when illuminated with 635 nm laser light at an angle of incidence of ${14}^\circ$. The phase contrast image (Fig. 5c) obtained within the contrast zone contains morphological detail absent in the corresponding bright field image, such as those highlighted within the dashed circles. The phase contrast produced is significantly enhanced compared to the relatively low contrast bright field image. Cellular thickness and local refractive index deviations are accented to discriminate the cells from their background.

As a baseline for comparison, a conventional differential interference contrast image was obtained of the same regions of the cells using the configuration outlined in the “Methods” section. The resultant image (Fig. 5d) also displays significant phase contrast with respect to the bright field image (Fig. 5a). The quality of the image is shown to be similar to the associated image obtained with the notch filter (Fig. 5b). Moreover, both images were taken in a microscope configuration, whose resolution was determined by its underlying optical components. Micrometre resolution was obtained as indicated by the scale bars in the images. As a result, features on the order of a micrometres were resolved by the system and revealed by the notch filter.

## Discussion

Inspired by the recent surge in interest towards meta-optical imaging, these results represent the first demonstration of biological image processing using a commercially available notch filter. The spatial frequency content of images was directly altered by the device in the object plane without needing to access the Fourier plane. The transfer function exhibited the required behaviour for high-pass filtering allowing edge detection, in addition to approximately linear regions permitting phase visualization through spatial differentiation. Rotating the filter enabled access to these regimes to offset the Fourier origin in $k$-space.

The contrast obtained in the images was significantly enhanced compared to the associated images without the notch filter. The regions of phase variation corresponded to intensity modulations in the filtered image. The quality of the images were competitive with those obtained in differential interference contrast microscopy. The latter utilizes interference effects to generate pseudo-three dimensional contrast in images along the orientation of a Wollaston prism. On the other hand, the notch filter generated comparable contrast determined by the direction of rotation without relatively expensive or bulky components. Zernike phase contrast microscopy similarly visualizes the phase of wavefields through spatial frequency filtering. However, this Fourier plane method cannot distinguish between positive and negative phase gradients of wavefields unlike the notch filter. Furthermore, artifacts are also produced in images if the phase gradients are too strong. In the case of the notch filter, phase gradients are shifted out of the contrast zone if they are too strong and hence do not appear in the image. However, artifacts can arise due to the thickness of the filter.

It was shown in the supplementary information (§S1.2) that the notch filter was capable of producing images similar to that obtained in Schlieren imaging by operating the near the edge of the contrast zone. The Schlieren method involves blocking either the positive or negative spatial frequencies of an image. Similarly, the notch filter suppresses spatial frequencies below the shifted $k$-space origin near the edge of the contrast zone, while those above are transmitted, thus approximately corresponding to the Schlieren method. In addition, operating within the suppression zone would lead to strong suppression of light from transmitting through the notch filter, associated with excluding low spatial frequencies as in dark field microscopy. This would produce dark field images of transparent samples that enhance features of strong phase variation, such as their edges, over a dark background. As a result, commercial notch filters offer a relatively cost efficient and readily available alternative for non-interferometric phase visualization through image processing without additional costly equipment. They are capable of producing images of competitive contrast and quality compared to other phase visualization methods, particularly useful in cases where these are unavailable or too expensive. The resolution and field of view of the images obtained with the filter are limited only by the optical system that they are used in.

Although the band-stop region was limited to within the visible band, various filters have been designed with coverage across the electromagnetic spectrum. For example, Yuan et al.^43^ presented a photonic filter employable at the absorption band of acetylene gas. Others include terahertz^44^, infrared^45^, microwave^46,47^ and commercial ultra-violet filters. These hold potential for phase imaging across the spectrum using the methods outlined in this paper, with applications in machine vision and biological imaging beyond the visible range. Live biological monitoring is possible with the notch filter dynamically revealing the structural dynamics of specimens through their phase variations using existing imaging sensors. Further applications include environmental monitoring and integration into portable conventional sensors to form phase-sensitive detectors. Incorporating notch filters into their detection planes would introduce spatial frequency selectivity and phase variations in otherwise indiscriminate, intensity-based devices.

Although phase contrast was produced by the notch filter, its behaviour in the contrast zone was not perfectly linear. Furthermore, its restricted numerical aperture limits the range of samples to which it could be applied. The large angular range of the suppression zone also restricts contrast to sufficiently sharp features. Not only is the range of samples limited, but it necessitates rotation of the notch filter. Finally, the theoretical and numerical modelling presented here did not include the non-negligible notch filter thickness, which can introduce perturbations into the beam path. This can lead to the appearance of aberrations such as those in the edge enhanced images in the supplementary information (§S1.2). However, the success of experiments performed here provide confidence that notch filters can be used as an alternative to existing phase contrast imaging methods. Moreover, custom thin film devices could be developed with reduced thicknesses to minimize aberrations and tailor the transfer function.

## Conclusion

In conclusion, this article has demonstrated real-time, all-optical, object-plane image processing using a commercial spectral notch filter. Spectroscopic measurements verified angle dispersive band-rejection necessary for high-pass spatial frequency filtering and phase contrast imaging. Edge detection was realizable through the suppression zone where unscattered field components were removed. Meanwhile, offsetting the transfer function to approximately linear regions within the contrast zone produced phase contrast images comparable to those obtained in differential interference contrast microscopy. Unstained biological samples, including a cell line derived from human cervical cancer, imposing otherwise invisible phase modulations were visualized by the filter. Therefore, notch filters offer a non-interferometric and off-the-shelf alternative to instantaneous phase visualization that can perform an equivalent role to other phase imaging methods. This has significant implications in label-free biomedical imaging^48^ including medical diagnostics, non-invasive micro-organism growth and dynamical monitoring. The results open developmental possibilities in extending to beyond the visible band and constructing monolithic spatial frequency-sensitive cameras for commercialization.

## Methods

### Simulations

Numerical simulations were performed in Python 3.9.5^49^ by implementing Eq. (1) with the transfer function of the notch filter. The experimentally measured modulation transfer function was combined with the interpolated phase response provided the manufacturer to model the optical response of the notch filter. The notch filter (Thorlabs NF633-25) used in this article was 3.5 mm thick and comprised tantalum pentoxide (Ta$_2$O$_5$) and silicon dioxide (SiO$_2$) thin films on a fused quartz substrate.

### Transfer function determination

White light from a fibre-coupled (Thorlabs SM600) halogen lamp (Ocean Insight HL-2000-FHSA) was collimated using a microscope objective (Nikon U Plan FL 20x/0.15NA). This light was polarized by a linear polarizer (Thorlabs LPVIS050-MP) to produce $p$- or $s$-polarized light. Circularly polarized light was produced by introducing an additional quarter waveplate (Thorlabs AQWP05M-600) before illuminating the notch filter. A lens (Thorlabs LA1068-A, $f={75}\,\hbox {mm}$) focused the transmitted light onto a fibre-coupled (Thorlabs M15L01) spectrometer (Ocean Insight QE6500). Measurements of the transmission spectra were made for angles of incidence ranging from ${-30}^\circ$ to ${+30}^\circ$ by incrementally rotating the filter by ${2}^\circ$.

### Phase contrast imaging

Fibre-coupled (Thorlabs SM600) 635 nm laser light (Thorlabs S1FC635) was collimated by a microscope objective (Nikon LU Plan 5x/0.15NA) and two lenses (Thorlabs LA1027-A $f={75}\,\hbox {mm}$ and LA1509-A $f={100}\,\hbox {mm}$). A linear polarizer (Thorlabs LPVIS050-MP) ensured that linearly polarized light illuminated a reflective spatial light modulator (Holoeye Pluto-2.1-VIS-001 LCOS-SLM). It programmed phase profiles of human red blood cells with a phase excursion of $6\pi /5$. The light reflected from the SLM passed through a quarter wave-plate (Thorlabs AQWP05M-600) to convert it to circular polarization. A lens (Thorlabs LA1433-A $f={150}\,\hbox {mm}$) and microscope objective (Olympus Plan N 20x/0.4NA) de-magnified the image onto the notch filter in its focal plane. The latter was longitudinally rotated by ${14}^\circ$ using a rotation mount to access the contrast zone. A microscope objective (Olympus Plan N 20x/0.4NA) and lens (Thorlabs LA1131-A $f={50}\,\hbox {mm}$) re-magnified and focused the filtered image onto a camera (Thorlabs DCC1645C).

### Phase contrast microscopy

Bright field microscopy was performed using white light from an inverted microscope (Nikon Ti-80i) without the notch filter. The illumination source was then replaced by fibre-coupled (Thorlabs SM600) 635 nm laser light (Thorlabs S1FC635) collimated by an objective (Olympus A4 4x/0.1NA), and a linear polarizer (Thorlabs LPVISC100-MP2) and quarter waveplate (Thorlabs AQWP05M-600) produced circularly polarized light. These optical components were contained within an optical cage mounted on an XYZ-stage to vary the angle of incidence. The notch filter was placed immediately beneath the sample on the microscope stage to perform phase contrast microscopy. A microscope objective (Nikon LU Plan 50x/LWD) collected the filtered light onto a camera (Andor Zyla sCMOS 4.2P). Finally, a differential interference contrast image was obtained using a microscope (Olympus BX60) with a microscope objective (Olympus Plan N 20x/0.4NA).

### Cell culture

The HeLa cells were provided by the Paul Gleeson laboratory of the Department of Biochemistry and Pharmacology and Bio21 Institute at the University of Melbourne. The cells were grown in Dulbecco’s modified Eagle’s medium (DMEM) (Lonza) supplemented with 10% heat inactivated bovine growth serum (Gibco), 1x Pen-Strep (Lonza) at ${37}^\circ \hbox {C}$ with 5% carbon dioxide. The cells were plated 24 h before fixation onto 35 mm glass bottom dishes, before being fixed with 4% paraformaldehyde for 15 min at room temperature and washed 3 times with phosphate-buffered saline (PBS).

## Supplementary Information

Supplementary Information.

## Data Availability

The datasets generated and/or analysed during the current study are available from the corresponding author on reasonable request.
