# Supplementary material for: Thin film notch filters as platforms for biological image processing
Source: Sci Rep. 2023 Mar 18;13:4494. doi: 10.1038/s41598-023-31528-5 (PMC10024701; doi:10.1038/s41598-023-31528-5)
Supplement: Supplementary file 1 — Supplementary Information. [file 41598_2023_31528_MOESM1_ESM.pdf]

Thin film notch filters as platforms for biological image processing - Supplementary information

Shaban B. Sulejman[1,*,+], Niken Priscilla[1,+], Lukas Wesemann[1], Wendy S. L. Lee[1,2], Jieqiong Lou[3], Elizabeth Hinde[3], Timothy J. Davis[1], and Ann Roberts[1]

[1]ARC Centre of Excellence for Transformative Meta-Optical Systems, School of Physics, The University of Melbourne, Victoria 3010, Australia.
[2]ARC Centre of Excellence for Transformative Meta-Optical Systems, Department of Electrical and Electronic Engineering, The University of Melbourne, Victoria 3010, Australia.
[3]School of Physics, The University of Melbourne, Victoria 3010, Australia.
[*]sulejmans@unimelb.edu.au
[+]These authors contributed equally to this work

ABSTRACT

Many image processing operations involve the modification of the spatial frequency content of images. Here we demonstrate object-plane spatial frequency filtering utilizing the angular sensitivity of a commercial spectral bandstop filter. This approach to all-optical image processing is shown to generate real-time pseudo-3D images of transparent biological and other samples, such as human cervical cancer cells. This work demonstrates the potential of non-local, non-interferometric approaches to image processing for uses in label-free biological cell imaging and dynamical monitoring.

S1 Experimental Methods & Results

S1.1 Optical Transfer Function

Spectroscopic measurements of the notch filter (Thorlabs NF633-25) were made using the experimental configuration depicted in Fig. 1 of the main article. Spectra were collected for p-, s- and circularly polarized light. The former two are given in Supplementary Fig. S1, while the latter is given in Fig. 2(a) of the main article. Each of these confirm suppression about the band-stop wavelength and its angular dispersion.

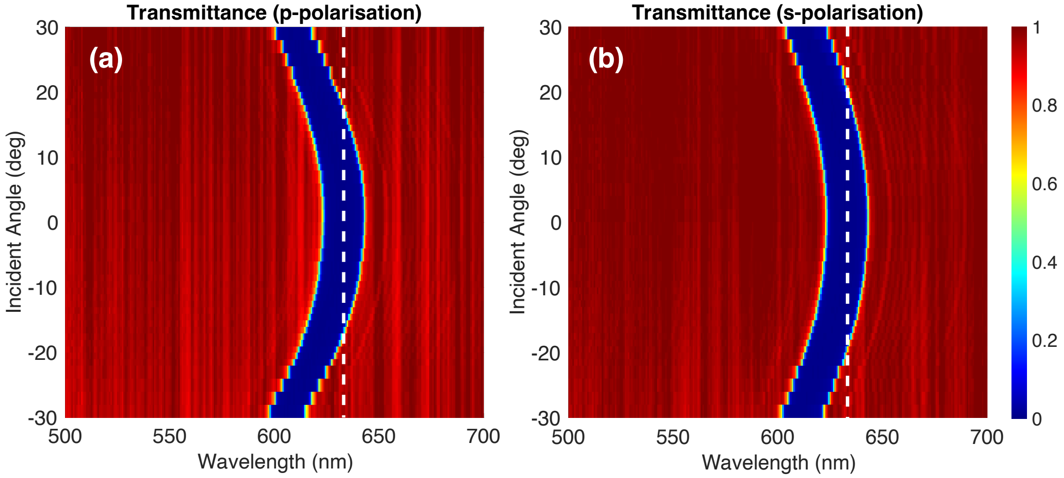

Figure S1. Transmission spectra of the notch filter obtained with p- and s-polarized light are given in (a) and (b), respectively. These exhibit similar behaviour with suppression around 633 nm (dashed lines) that blue-shifts with increasing incident angle.

S1.2 Edge Detection

All-optical image processing was experimentally performed to demonstrate edge detection with the notch filter. As depicted in Supplementary Fig. S2, fibre-coupled (Thorlabs SM600) 635 nm laser light (Thorlabs S1FC635) was collimated by a microscope objective (Nikon LU Plan 5x/0.15NA) and two lenses (Thorlabs LB1901-B $f = 75$ mm and LB1761-B $f = 25.4$ mm). Light reflected from a mirror was projected onto the the largest regions of a USAF resolution test target (Thorlabs R2L2S1N1 NBS 1963A) using a microscope objective (Olympus U Plan FL 20x/0.50NA) and a lens (Thorlabs LA1509-A $f = 100$ mm). Upon reflection from a mirror, a lens (Thorlabs LA1433-A $f = 150$ mm) and a microscope objective (Nikon U Plan FL 20x/0.4NA) projected a collimated image onto the notch filter in the focal plane of the microscope objective. The filtered image was re-magnified by a microscope objective (Nikon LU Plan 50x/0.55NA) and focused onto a camera (Thorlabs DCC1645C) by a lens (Thorlabs LA1131-A $f = 50$ mm).

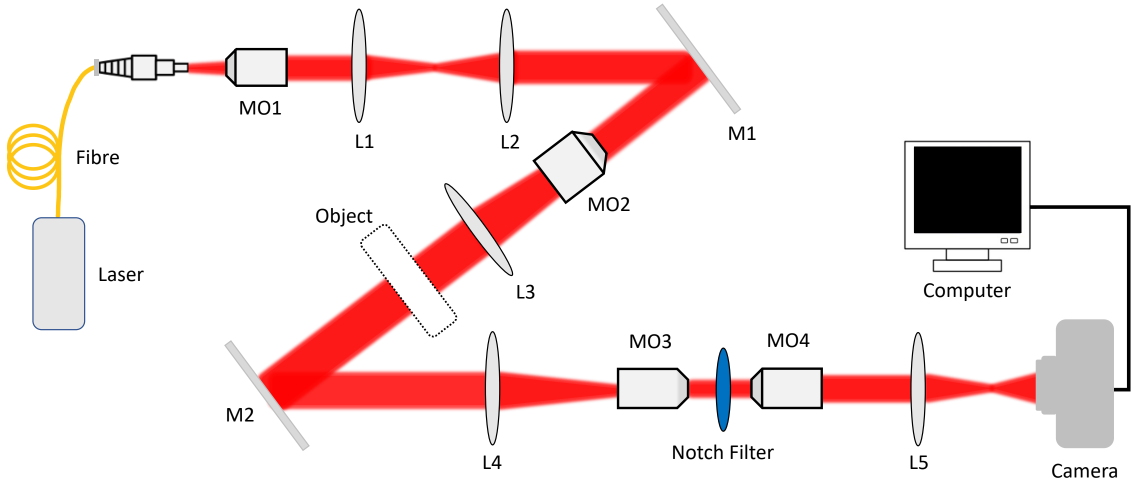

Figure S2. A schematic of the experimental configuration for edge detection with a notch filter is shown. Here L, MO and M each denote lenses, microscope objectives and mirrors, respectively. The schematic is not to scale

Given in Figs. S3(a) and (b), the simulated and experimental results demonstrate edge enhancement produced by the notch filter. The uniform intensity regions associated with low spatial frequency components were removed, leaving only the edges. This is supported by line profiles given in Supplementary Fig. S3(c). Some slight asymmetries are present in the experimental edge-enhanced image owing to the rotations of the filter and its thickness.

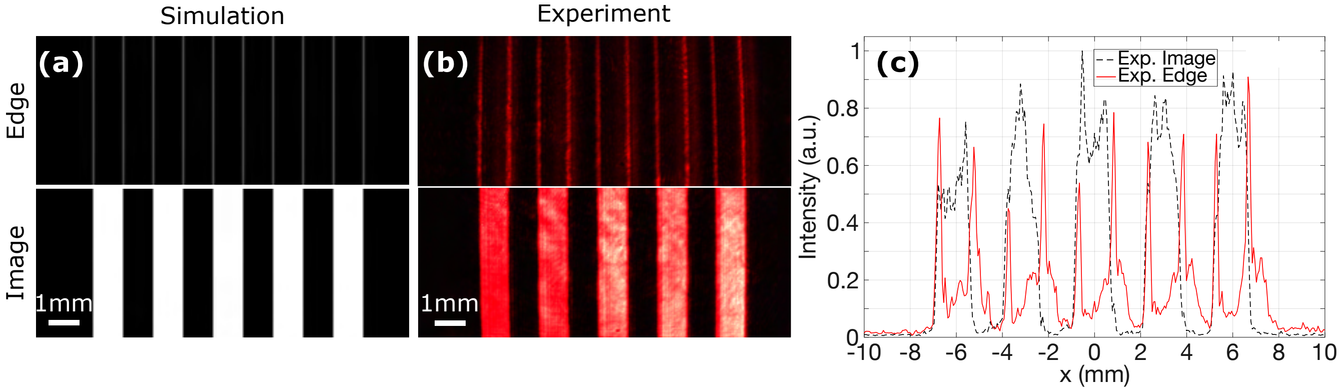

Figure S3. Edge-enhanced images of USAF test target bars overlayed on their unfiltered images are given in (a) and (b). Line profiles in (c) depict suppression within the uniform regions of the bars.
